# Pemphigus vulgaris associated with nasoseptal perforation, ocular conjunctival herpes infection and milia formation^☆^

**DOI:** 10.1016/j.abd.2021.09.019

**Published:** 2023-03-21

**Authors:** Sebastian Vernal, Roberto Bueno-Filho, Takashi Hashimoto, Ana Maria Roselino

**Affiliations:** a Dermatology Division, Department of Clinical Medicine, Faculdade de Medicina de Ribeirão Preto, Universidade de São Paulo, Ribeirão Preto, SP, Brazil; b Department of Dermatology, Kurume University School of Medicine, and Kurume University Institute of Cutaneous Cell Biology, Fukuoka, Japan

*Dear Editor,*

We report a 60-year-old woman who was diagnosed with pemphigus vulgaris (PV) associated with uncommon presentations, i.e., nasal mucosal involvement with septal perforation, ocular conjunctival involvement of herpes simplex virus (HSV) infection, and milia on the re-epithelialized skin. We will discuss each association based on the results of our laboratory examinations.

Clinical examination revealed erosive skin lesions mainly on the face, trunk, and limbs, as well as mucosal lesions on the tongue, gingivae, and palate (Fig. 1A), and hyperemia on the left conjunctiva. Erosions and crusts in the nasal mucosa, and anterior nasoseptal perforation were also detected. Additionally, milia were observed on the skin of the face and shoulders, which were previously affected by PV (Fig. 1B).

**Figure 1:**
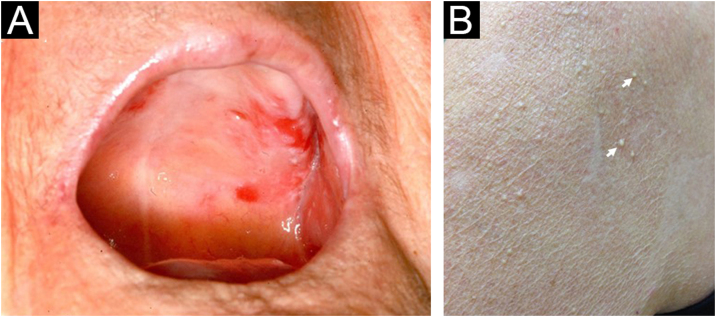
Clinical features of the present case. (A) Erosions on the soft and hard palate. (B) Milia seen over previously erosive PV lesions on the shoulder (arrows).

Histopathology showed suprabasal acantholysis for biopsies taken from the chest and from the nasal septum (Fig. 2A). IgG deposition on keratinocyte cell surfaces was detected on direct immunofluorescence (DIF) using Tzanck smears from oral mucosa (Fig. 2B), which suggested PV. Anti-herpes simplex virus (HSV)-1 antibody (Abcam, Cambridge, USA) on DIF using scraped smears showed negative staining in oral mucosa but positive nuclear staining in ocular conjunctiva (Fig. 2C), while immunohistochemistry using nasal mucosa biopsy showed negative HSV staining. Polymerase-chain-reaction for *Leishmania* sp., *Mycobacterium* sp., *M*. *tuberculosis* and *M*. *leprae*, and fungal culture from nasal mucosa samples were negative.

**Figure 2:**
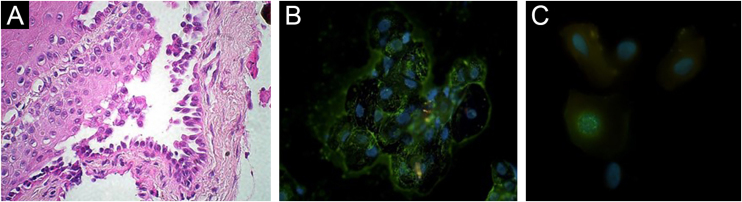
Histopathological and immunofluorescence findings. (A) Suprabasal acantholysis in biopsy taken from nasal mucosa (Hematoxylin & eosin, ×63). (B) Positive reactivity in oral mucosal cell surfaces with FITC-conjugated anti-human IgG antibody using Direct Immunofluorescence (DIF) on Tzanck smears taken from oral mucosae (×63). (C) DIF using ocular conjunctival scraped smears with FITC-conjugated anti-HSV-1 antibody showing positive nuclear staining (×100).

Antinuclear antibody, HIV and VDRL serologies were negative. indirect immunofluorescence (IIF) using a normal human skin section detected IgG anti-keratinocyte cell surface antibodies. IIF on 1M-NaCl-split-skin was negative in the basement membrane zone.

ELISA (MBL, Japan) was positive for desmoglein (Dsg) 1 (112.7 U/mL) and Dsg3 (39.3 U/mL) (cut-off 20 U/mL), but negative for BP180 (4.21 U/mL) and BP230 (1.51 U/mL) (cut-off 9 U/mL). Immunoblotting analyses using normal human epidermal and dermal extracts, recombinant proteins of BP180 NC16a and C-terminal domains, concentrated culture supernatant of HaCaT cells, and purified human laminin – 332 were all negative.

The patient underwent three monthly cycles of pulse therapy (dexamethasone and cyclophosphamide), followed by oral prednisone 12.5 mg/day, and cyclophosphamide 50 mg/day. Acyclovir was also given. Skin lesions quickly disappeared. All the mucosal lesions gradually improved.

Nasal mucosal involvement in PV has rarely been reported^1^. In this case, histopathology showed suprabasal acantholysis in nasal mucosa biopsy. Collagen disease and infections of HIV, syphilis, HSV, leishmaniasis, leprosy, and fungus were excluded^2^. We suppose that the nasoseptal perforation was attributed secondarily to repetitive traumatic manipulation for PV-induced nasal mucosal crusts.

DIF on the Tzanck smear suggested HSV-1 infection for the left conjunctival lesion, and the conjunctival lesions improved after acyclovir treatment. HSV-1 infection was occasionally observed on the oral mucosa^3^, but not on the ocular conjunctiva, in PV.

Finally, our patient showed milium formation in skin previously affected by PV. Therefore, we also performed various IIF, ELISA, and immunoblotting analyses, which excluded the diagnoses of epidermolysis bullosa acquisita and bullous pemphigoid, the diseases commonly developing milia^4^.

In conclusion, although PV is a prevalent disease in Southeastern Brazil^5^, this is the first PV case with nasoseptal perforation, conjunctival herpetic infection and milium-associated features.

## Financial support

This study was partially supported by FAPESP (Fundação de Amparo à Pesquisa do Estado de São Paulo), process number 2010/51729-2, and by FAEPA (Fundação de Apoio ao Ensino, Pesquisa e Assistência). SV received a PhD scholarship from CAPES (Coordenação de Aperfeiçoamento de Pessoal de Nível Superior).

## Authors' contributions

Sebastian Vernal: Has contributed with collection and interpretation of data, writing the manuscript, effective participation, literature review, final approval of the final version of the manuscript.

Roberto Bueno-Filho: Has contributed with collection and interpretation of data, effective participation, participation of therapeutic conduct of the studied cases, final approval of the final version of the manuscript.

Takashi Hashimoto: Has contributed with collection and interpretation of data, effective participation, writing the manuscript, final approval of the final version of the manuscript.

Ana Maria Roselino: Has contributed with the study concept and design, collection, and interpretation of data, writing the manuscript, effective participation, literature review, participation of therapeutic conduct of the studied cases, final approval of the final version of the manuscript.

## Conflicts of interest

None declared.
